# Assessment of Hospital Emergency Department Response to Potentially Infectious Diseases Using Unannounced Mystery Patient Drills — New York City, 2016

**DOI:** 10.15585/mmwr.mm6636a2

**Published:** 2017-09-15

**Authors:** Mary M.K. Foote, Timothy S. Styles, Celia L. Quinn

**Affiliations:** ^1^Bureau of Healthcare System Readiness, Office of Emergency Preparedness and Response, New York City Department of Health and Mental Hygiene, New York City, New York; ^2^Field Services Branch, Division of State and Local Readiness, Office of Public Health Preparedness and Response, CDC.

Recent outbreaks of infectious diseases have revealed significant health care system vulnerabilities and highlighted the importance of rapid recognition and isolation of patients with potentially severe infectious diseases. During December 2015–May 2016, a series of unannounced “mystery patient drills” was carried out to assess New York City Emergency Departments’ (EDs) abilities to identify and respond to patients with communicable diseases of public health concern. Drill scenarios presented a patient reporting signs or symptoms and travel history consistent with possible measles or Middle East Respiratory Syndrome (MERS). Evaluators captured key infection control performance measures, including time to patient masking and isolation. Ninety-five drills (53 measles and 42 MERS) were conducted in 49 EDs with patients masked and isolated in 78% of drills. Median time from entry to masking was 1.5 minutes (range = 0–47 minutes) and from entry to isolation was 8.5 minutes (range = 1–57). Hospitals varied in their ability to identify potentially infectious patients and implement recommended infection control measures in a timely manner. Drill findings were used to inform hospital improvement planning to more rapidly and consistently identify and isolate patients with a potentially highly infectious disease.

Exercises were designed in accordance with the U.S. Department of Homeland Security Exercise and Evaluation Program (*1*). Scenarios were developed in collaboration with a stakeholder advisory group and consisted of a person simulating a patient entering the ED and reporting recent fever and either 1) respiratory symptoms and recent travel to the Middle East (i.e., possible MERS) or 2) a rash after traveling to Europe (i.e., possible measles). A red maculopapular measles-like rash was simulated on the neck or upper extremities of the person in the role of the measles patient using a commercially available moulage kit (Figure 1). Based on previously provided ED guidance (*2*), the expectation was that once the patient was identified as being at high risk for having a communicable disease with a potential for respiratory transmission, he or she would be asked to don a mask and would be placed into an airborne infection isolation room.

**FIGURE 1:**
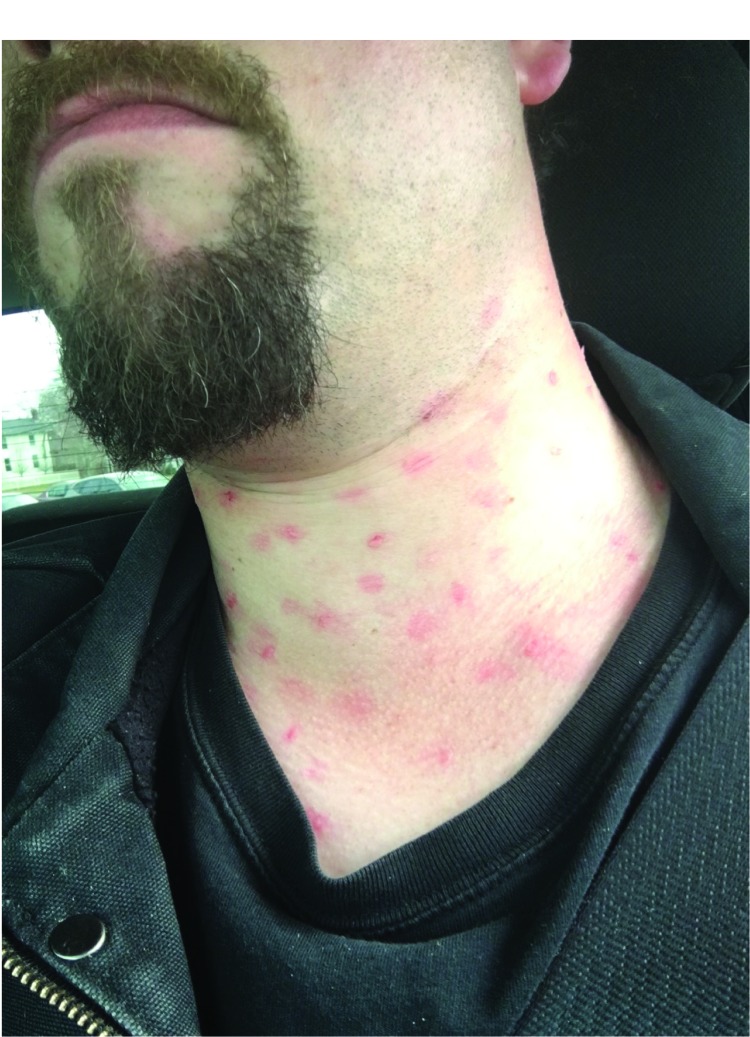
Patient actor displaying moulage-simulated measles rash during mystery patient drills — New York City, December 2015–May 2016 Photo/New York City Department of Health and Mental Hygiene

All 50 New York City hospitals with emergency departments that participate in the 911 system and receive Hospital Preparedness Program funding through the U.S. Department of Health and Human Services Office of Assistant Secretary of Preparedness and Response were offered the opportunity to participate in the program; 49 agreed to take part. Exercises were conducted with a simulated patient (who served as the exercise controller), an evaluator, and up to two hospital employees (serving as trusted agents) who helped coordinate the visit. No other hospital staff members were informed of the drill. The controller entered the ED unannounced, and, when prompted by ED staff members, reported signs or symptoms consistent with the exercise scenario. The evaluator entered the ED separately with one of the trusted agents and remained in the ED during the exercise to collect data using a standardized exercise evaluation guide. The controller ended the exercise after the initial evaluation by a health care provider. Exercises were terminated and considered failed if ED wait time exceeded 30 minutes without triage. The following outcomes were evaluated: 1) compliance with key infection control measures, including staff member hand hygiene, appropriate use of personal protective equipment (PPE), and infection prevention signage; 2) association between screening interventions (e.g., travel screening) and implementation of infection control measures; and 3) key quantitative measures including time from entry of the patient until triage, until donning a mask, and until isolation. The exercise was considered successful (i.e., “passed”) if the patient was given a mask and isolated from other patients and staff members. At the conclusion of the drill, exercise staff members facilitated a debriefing with all the drill participants including the facility trusted agents. Descriptive analyses and chi-square tests for association were performed using statistical software with p-values <0.05 considered to be statistically significant. Variable specific analyses of times excluded drills with missing time stamp data.

Forty-nine New York City hospitals participated in 95 (53 measles, 42 MERS) drills during December 2015–May 2016. Overall, 76 (80%) patients were asked about recent fevers, and 81 (85%) were asked about recent travel. Questions about a rash or unusual skin lesions or respiratory symptoms were asked of 47 (50%) and 69 (68%) patients, respectively. Overall, 84 (88%) patients were given a mask, including 45 (85%) patients in the measles scenarios and 39 (93%) patients in the MERS scenarios.

Among all 95 drills, 74 (78%) passed, including 35 (83%) of 42 MERS scenarios and 39 (74%) of 53 measles scenarios (p = 0.3). Similarly, there were no significant differences in the percentage of simulated MERS and measles patients who received a mask (93% versus 85%) or were isolated (83% versus 77%) (Figure 2). Nineteen (39%) of 49 hospitals failed at least one drill. Masking and isolation occurred in 88% (71 of 81) drills when travel history was obtained, compared with only 21% (3 of 14) drills when such history was not obtained (p<0.001). The median time from patient entry to triage was 1 minute for both scenarios (Table). The median time from patient entry to masking was 1 minute in the measles scenario and 2 minutes in the MERS scenario, and from patient entry to isolation was 8 minutes in the measles scenario and 11 minutes in the MERS scenario.

**FIGURE 2:**
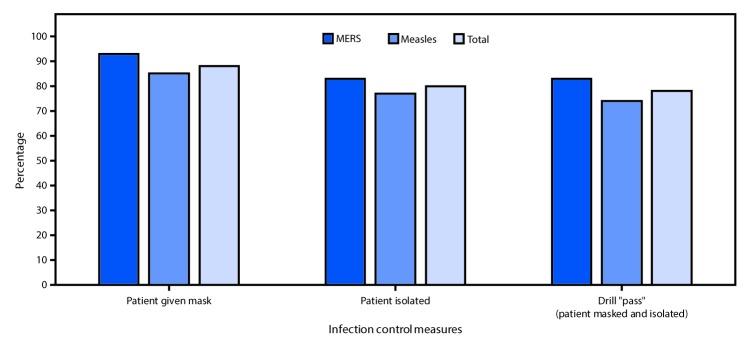
Adherence to mask use and isolation protocols and drill pass rate* in 95 mystery patient drills, by scenario^†^ — 49 New York City emergency departments, December 2015–May 2016 **Abbreviation**: MERS = Middle East Respiratory Syndrome * “Patient” asked to don a mask and isolated from other patients and staff members. **^†^** Simulation drill, with “patient” describing signs and symptoms and providing travel history consistent with either possible MERS or measles.

**TABLE:** Median intervals from patient entry to implementation of specific infection control measures* in simulated measles (N = 53) and MERS (N = 42) scenarios — 49 New York City hospital emergency departments, December 2015–May 2016

Infection control measure	Measles scenarios		MERS scenarios		All scenarios	
	No. scenarios	Minutes, median (range) to implement	No. scenarios	Minutes, median (range) to implement	No. scenarios	Minutes, median (range) to implement
Entry to triage	52	1 (0–26)	41	1 (0–30)	93	1 (0–30)
Entry to masking	45	1 (0–26)	39	2 (0–47)	84	1.5 (0–47)
Entry to isolation	41	8 (1–41)	35	11 (1–57)	76	8.5 (1–57)

**Abbreviation**: MERS = Middle East Respiratory Syndrome.

* Drills with missing time stamps were excluded.

Assessment of other infection control practices found that 36% of staff members performed personal hand hygiene and 16% of staff members instructed patients to perform hand hygiene. In the 76 (80%) drills that resulted in the patient being isolated, precaution signage was posted outside the patient’s airborne isolation room of 53 (70%), and staff members used recommended PPE when entering these rooms in 56 (74%) drills.

## Discussion

EDs and their associated waiting areas have been shown to facilitate the transmission of infections, such as measles and severe acute respiratory syndrome, to patients and health care workers, leading to spread within hospitals and surrounding communities (*3*,*4*). This mystery patient drill program provided an opportunity to examine real-world implementation of infectious disease-related screening and isolation of potentially high-risk patients in EDs across New York City. It also provided a reasonable baseline for expectations of ED staff member practices regarding control of highly infectious diseases at this entry point to the hospital system. Based on these findings, performance goals of 1 minute from entry to masking and 10 minutes from entry to isolation will be adopted for evaluating similar drills in the future. In addition, the overall median time from entry to isolation achieved in this study (8.5 minutes) is comparable to times achieved in an earlier Ebola drill analysis (9 minutes) (*5*).

Although the majority of drills were completed successfully by masking and isolating the patient, approximately 40% of hospitals failed at least one drill, and there was considerable variation in the length of time each hospital took to perform these steps. It is possible that measles cases were recognized to be an infectious risk more quickly, as the rash was a clearer objective finding. However, the higher percentage of mask provision and patient isolation in MERS scenarios suggests that a history of travel to the Middle East might be more recognizable as a high-risk exposure than history of travel to Germany in the measles scenario; it was noted on multiple drill reports that staff members were unsure if travel to Europe constituted a risk. The finding that masking and isolation occurred significantly more frequently in situations where a travel history had been elicited suggests that routinely inquiring about recent travel could prevent exposures to infectious patients at critical entry points to the health care system.

Another important finding was suboptimal adherence to key infection control practices, including hand hygiene (36%), PPE use (74%), and posting of isolation signage (70%), highlighting the need for routine competency-based infection-control training programs.

Simulated patient exercises have been demonstrated to be effective tools to evaluate hospital emergency plans (*6*), and studies have validated their use for testing health care system preparedness for communicable diseases of public health concern, including Ebola, avian influenza, inhalation anthrax, and smallpox (*6*–*10*). This is the first report describing the use of unannounced mystery patient drills to test ED preparedness for MERS and measles. Whereas other studies have described specific infection-control interventions, such as patient masking (*7*), isolation (*9*), and risk-factor screening (*8*), this study is unique in its use of drills to capture both key temporal measures and staff member compliance with multiple infection control practices.

The findings in this report are subject to at least two limitations. First, exercise evaluation was limited to items that were under direct control of the staff members who participated in the drill, the controller, and the evaluator. Factors such as ED patient volume and staffing levels could potentially influence performance on a given day, but these were not evaluated. Second, controllers were not able to objectively present all signs of illness (e.g., fever, chills), and the moulage used to simulate a measles rash might have been misleading or unconvincing, although this information was not captured in the drill reports.

Unannounced mystery patient drills were successfully used to evaluate communicable disease response capabilities in the acute care setting in 49 New York City hospital EDs. As part of this program, a toolkit was developed to help hospitals carry out similar infectious disease drills to test protocols and identify areas for improvement. Use of standardized scenarios, evaluation guides, and reporting templates can assist public health officials in assessing system-wide capabilities and gaps to guide interventions, and inform development of training resources to improve health care facility readiness at a critical point of entry into the health care system. The toolkit is available at http://on.nyc.gov/IDPrep.

**Summary**

**What is already known about this topic?**

Recent infectious disease epidemics highlight the importance of rapid recognition and isolation of patients with severe infectious diseases. Unannounced mystery patient drills have been used in the health care setting to evaluate protocols and staff members’ ability to identify and manage potentially infectious patients.

**What is added by this report?**

Ninety-five mystery patient drills were conducted in 49 New York City hospital emergency departments to assess responsiveness to patients with potentially severe infections. The times required to perform patient masking and isolation were evaluated; overall, patients were masked and isolated in 78% of drills. Masking and isolation occurred significantly more frequently when travel history was obtained (88%) than when it was not (21%). Overall, the median time from patient entry to masking was 1.5 minutes (range = 0–47 minutes) and from entry to isolation was 8.5 minutes (range = 1–57).

**What are the implications for public health practice?**

A toolkit was developed to support health care facilities and health departments conduct similar drills to identify areas for improvement and enhance readiness at a critical point of entry into the health care system. This toolkit could be useful for other jurisdictions.
